# Myocarditis Attributable to Monkeypox Virus Infection in 2 Patients, United States, 2022

**DOI:** 10.3201/eid2812.221276

**Published:** 2022-12

**Authors:** Guillermo Rodriguez-Nava, Peter Kadlecik, Thomas D. Filardo, David L. Ain, Joseph D. Cooper, David W. McCormick, Bryant J. Webber, Kevin O’Laughlin, Brett W. Petersen, Supriya Narasimhan, Harleen K. Sahni

**Affiliations:** Stanford University School of Medicine, Stanford, California, USA (G. Rodriguez-Nava);; Mid-Atlantic Permanente Medical Group, Rockville, Maryland, USA (P. Kadlecik);; Centers for Disease Control and Prevention, Atlanta, Georgia, USA (T.D. Filardo, D.W. McCormick, B.J. Webber, K. O’Laughlin, B.W. Petersen);; Mid-Atlantic Permanente Medical Group, Washington DC, USA (D.L. Ain);; Santa Clara Valley Medical Center, San Jose, California, USA (J.D. Cooper, S. Narasimhan, H.K. Sahni)

**Keywords:** monkeypox, myocarditis, immunocompetent, orthopox, viruses, sexually transmitted infections, unvaccinated, vaccine-preventable diseases, United States

## Abstract

We report 2 immunocompetent and otherwise healthy adults in the United States who had monkeypox and required hospitalization for viral myocarditis. Both patients were unvaccinated against orthopoxviruses. They had shortness of breath or chest pain and elevated cardiac biomarkers. No immediate complications were observed. They were discharged home after symptoms resolved.

Monkeypox is a zoonotic orthopoxvirus that is endemic to West and Central Africa and has caused sporadic outbreaks elsewhere (*1*,*2*). On July 23, 2022, the World Health Organization declared the 2022 monkeypox outbreak a Public Health Emergency of International Concern (*3*). Human monkeypox manifests as a viral syndrome, typically involving prominent lymphadenopathy and characteristic skin lesions (*2*,*4*). However, severe manifestations have been reported in children, pregnant women, and immunocompromised persons (*1*,*4*,*5*).

## The Study

**Figure 1:**
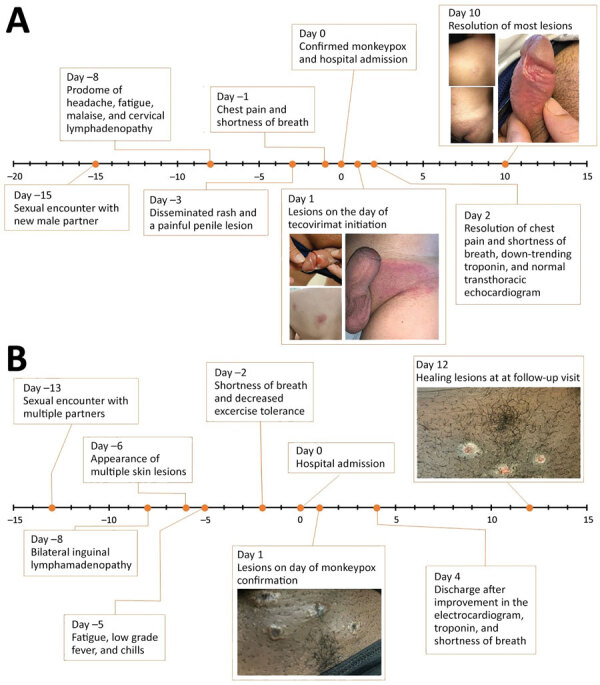
Timeline of events for 2 immunocompetent patients with monkeypox complicated by myocarditis, United States. A) A healthy 32-year-old man (patient 1) had chest pain and shortness of breath 7 days after a prodrome of headache, fatigue, malaise, and cervical lymphadenopathy and 2 days after the rash. Symptoms resolved after 10 days of illness onset and 1 day after initiation of tecovirimat. The patient received supportive care only for myocarditis. B) A healthy 37-year-old man (patient 2) had shortness of breath and decreased exercise tolerance 6 days after illness onset with bilateral inguinal lymphadenopathy and 4 days after the rash. Shortness of breath improved after 12 days of illness onset, and exercise tolerance normalized after 20 days. The patient received supportive care only for both monkeypox and myocarditis.

Both patients in this study provided informed consent for publication of deidentified medical information. Patient 1 was a healthy 32-year-old man who sought care at a hospital for his diagnosis of monkeypox. He reported having a sexual encounter with a new male partner 15 days earlier. Seven days after that encounter, he had onset of a viral illness with cervical lymphadenopathy, followed by a disseminated rash and a painful penile lesion. Two days before his hospital visit, a nonvariola orthopoxvirus DNA PCR test on a skin lesion specimen was positive. In the hospital, the patient reported ongoing chest pain and dyspnea for 1 day (Figure 1). He reported prior treatment for syphilis. He did not report recent SARS-CoV-2 vaccination or infection and was unvaccinated for smallpox. Physical examination revealed multiple erythematous vesiculopapular and pustular lesions with erythematous borders, left inguinal lymphadenopathy, and ulceration at the base of the glans penis.

Laboratory results were notable for a nonreactive HIV by p24 antigen testing, negative HIV and hepatitis C PCR tests on serum samples, and a rapid plasma reagin titer of 1:2. Cardiac biomarkers revealed an elevated high-sensitivity troponin T (165 ng/L [reference <22 ng/L]) and elevated levels of N-terminal prohormone B-type natriuretic peptide (1,258 pg/mL [reference <450 pg/mL]). Electrocardiogram showed normal sinus rhythm, and chest radiograph results were unremarkable. A nasopharyngeal respiratory viral panel was unrevealing. C-reactive protein was 0.5 mg/dL (reference <0.5 mg/dL), and erythrocyte sedimentation rate was 11 mm/h (reference <15 mm/h). PCR on serum samples was negative for enterovirus and adenovirus. SARS-CoV-2 nucleocapsid antibody results were negative. Serologic test results for parvovirus, cytomegalovirus, Epstein–Barr virus, coccidioidomycosis, and human herpesvirus 6 were negative.

The patient was admitted for suspected myocarditis and started on oral tecovirimat for treatment of monkeypox and doxycycline (because of penicillin allergy) for syphilis of unknown latency. He received no specific treatment for myocarditis given the rapid resolution of symptoms and normalization of troponin levels.

On hospital day 2, echocardiography showed an ejection fraction of 69% (reference 50%–75%) without wall motion abnormalities. By hospital day 6, the high-sensitivity troponin decreased to 11 ng/L from the initial peak of 165 ng/L. The skin and penile lesions had improved, with crusting and exfoliation of >80% of the lesions; however, the patient required prolonged hospitalization to maintain strict isolation. On hospital day 10, the only active lesion was a small penile ulcer in the process of epithelialization, and the patient was discharged to home with isolation precautions and instructions to complete a 14-day course of oral tecovirimat.

Patient 2 was a previously healthy 37-year-old man evaluated in the hospital for rash, fever, dyspnea, and decreased exercise tolerance 13 days after a sexual encounter with multiple partners. Five days after that encounter, he had onset of bilateral inguinal lymphadenopathy, followed by multiple skin lesions in both arms and a lesion at the base of the penis 2 days later. The next day, he had fatigue, low-grade fever, and chills. Two days before he sought care at the hospital, he had difficulty breathing and decreased exercise tolerance without chest pain. He reported dyspnea after climbing a single flight of stairs, a marked decrease from his baseline (Figure 1). He had a history of treated syphilis, was taking HIV preexposure prophylaxis, and reported that his mother died at age 40 from coronary artery disease. He did not report recent SARS-CoV-2 vaccination or infection and was unvaccinated for smallpox. 

**Figure 2:**
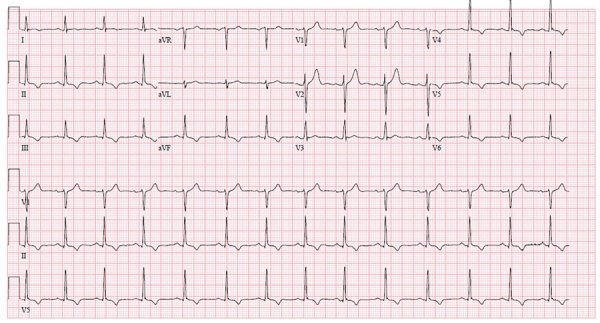
An electrocardiogram of a healthy 37-year-old man (patient 2) with monkeypox, shortness of breath, and decreased exercise tolerance shows normal sinus rhythm with T wave inversions in the inferior and anterolateral leads.

Physical examination showed multiple skin lesions with central umbilication in the lower pubic and inguinal areas with smaller vesicular lesions on upper extremities. Laboratory results were notable for an elevated serum troponin I (0.35 ng/mL [reference <0.07 ng/mL]); serial measurements at 4 and 8 hours were stable (0.34 and 0.39 ng/mL, respectively). B-type natriuretic peptide level was 49 pg/mL (reference <100 pg/mL). An electrocardiogram demonstrated normal sinus rhythm, with T wave inversions in the inferior and anterolateral leads (Figure 2). Subsequent tracings showed improvement in the repolarization abnormality. Echocardiography showed normal biventricular size and systolic function with normal regional wall motion, and diastolic indices were age-appropriate. 

The diagnosis of monkeypox was confirmed by nonvariola orthopoxvirus PCR from skin lesion specimens. Additional testing showed negative HIV by p24 antigen testing, baseline rapid plasma reagin titer of 1:1 (consistent with treated syphilis), and a negative SARS-CoV-2 PCR result. Additional investigations for other causes of myocarditis were deferred.

The patient remained hospitalized for 4 days. Dyspnea improved on day 3 and resolved by day 4; cardiac enzymes normalized. The patient received supportive care without directed therapy for monkeypox or myocarditis. After improvement, he was discharged with isolation precautions.

Although clade testing results were unavailable, these patients were presumed to have clade IIb infection given the epidemiology of the ongoing global monkeypox outbreak and their lack of an epidemiologic link to clade I (i.e., no relevant travel history or animal exposures). Although monkeypox-associated myocarditis was considered the most likely etiology for both patients, given the temporal relationship, we could not completely confirm the diagnosis with histopathologic tests or exclude other potential etiologies, including viral co-infections.

## Conclusions

The clinical course of human monkeypox is milder than that of smallpox in immunocompetent hosts (*6*). However, severe complications have been identified, including pneumonitis, encephalitis, eyesight-threatening keratitis, secondary bacterial infections, acute kidney injury, and myocarditis (*1*,*2*,*4*–*6*). Thornhill et al. (*5*) recently reported 2 cases of self-limited myocarditis in patients with monkeypox that resolved within 7 days without major complications; 1 patient had a history of HIV with a normal CD4 cell count. Similarly, the patients in our report improved 10–12 days after illness onset; 1 patient received tecovirimat, an inhibitor of the orthopoxvirus VP37 envelope-wrapping protein that prevents the formation of egress-competent enveloped virions and has been shown to decrease circulating viral DNA in a nonhuman primate model (*7*).

Many viruses have been associated with myocarditis (*8*,*9*). The most common pathophysiology of viral myocarditis is lymphocytic myocarditis associated with myonecrosis that occurs 10–14 days postinfection; illness can be either self-limiting or result in fulminant myocarditis. In some cases, viral myocarditis can progress to a noninfectious chronic phase, characterized by myocardial fibrosis, cardiac dysfunction, and dilated cardiomyopathy (*9*).

Myocardial involvement of orthopox infections was initially reported when myocarditis was observed after smallpox vaccination with replicating vaccinia-based vaccines in young military recruits (*10*). The pathophysiology of orthopox-induced myocarditis remains unknown. However, an autoimmune-mediated phenomenon has been postulated because of the absence of direct viral infection of the myocytes observed on histopathologic examination of samples from vaccinees with myocarditis (*10*). Most cases are mild and self-limited; major sequelae, such as dilated cardiomyopathy, are rare (*11*).

Hemorrhagic smallpox, the most severe manifestation of variola major, is characterized by rapid onset fever, rash, and disseminated intravascular coagulation. Anatomopathologic studies in hemorrhagic smallpox patients showed myocardial and endocardial hemorrhages (*12*). In a macaque model of hemorrhagic smallpox, histopathologic tests at day 6 or 7 postexposure showed acute lymphohistiocytic myocarditis with myocardiocyte degeneration and hemorrhage, primarily driven by direct viral myocardial injury and mediated by CD14 monocytes, chemotactic cytokines, and interleukin 6 (*12*). Therefore, we hypothesize that direct myocardial infiltration associated with monkeypox viremia may also result in myocarditis and that antiviral agents could play a role in treatment.
